# Does overnight memory consolidation support next-day learning?

**DOI:** 10.1016/j.cognition.2025.106241

**Published:** 2025-11

**Authors:** Anna á V. Guttesen, Marcus O. Harrington, M. Gareth Gaskell, Scott A. Cairney

**Affiliations:** a Department of Psychology, University of York, UK; b Wellcome Centre for Integrative Neuroimaging, Nuffield Department of Clinical Neurosciences, University of Oxford, UK; c School of Psychology, University of East Anglia, UK; d York Biomedical Research Institute, University of York, UK

**Keywords:** 12–12 design, Encoding capacity, Memory consolidation, Sleep, Paired-associates learning, Preregistered, Open data

## Abstract

Sleep supports memory consolidation and next-day learning. The Active Systems model of consolidation proposes that sleep facilitates a shift in the memory retrieval network from hippocampus to neocortex in service of long-term storage. Accordingly, overnight consolidation may support efficient next-day learning. We tested this hypothesis across two preregistered behavioural experiments. In both experiments, participants learned a set of word pairs and recall was assessed before and after a 12-h delay containing overnight sleep or daytime wakefulness. Participants then learned and were immediately tested on a new set of word pairs. Word pair retention was better after the delay of sleep than wakefulness, suggesting a benefit of sleep for memory consolidation, but there was no sleep-related learning advantage for the new set of word pairs. Sleep-associated consolidation was not associated with next-day learning in our preregistered analyses, although a significant positive relationship with learning did emerge in an exploratory analysis that accounted for performance at pre-sleep recall. Taken together, our findings provide exploratory evidence that overnight consolidation may be linked to new learning, with pre-sleep retrieval performance influencing the magnitude of this relationship.

## Highlights

• Memory retention was tested over a delay of overnight sleep or daytime wakefulness.
• Ability to learn new information was also tested after the delay.
• Sleep (vs wakefulness) benefitted memory retention but not new learning.
• Overnight memory retention was associated with new learning in exploratory analysis.

## 1 Introduction

How do we form new memories without overwriting existing ones? The dilemma between stability and plasticity was addressed by the Standard Model of Consolidation (Marr, Willshaw, & McNaughton, 1971; McClelland, McNaughton, & O'Reilly, 1995; Squire, Cohen, & Nadel, 1984), which describes two distinct memory stores: a hippocampal short-term store and a neocortical long-term store. Episodic declarative memories (i.e., memories for events that can be consciously brought to mind) are thought to be encoded into both the hippocampus and neocortex, with the hippocampus supporting retrieval through the binding of disparate neocortical memory traces (Frankland & Bontempi, 2005). Over subsequent days and weeks, memories are repeatedly reactivated and cortico-cortical connections are gradually strengthened, allowing memory retrieval to occur in the absence of hippocampal input. This systems-level shift in retrieval dependency, according to the standard model, allows prior experiences to become integrated into long-term memory and ensures that the hippocampus has the capacity to continue to encode new information.

Sleep supports declarative memory consolidation (Ashton & Cairney, 2021; Ashton, Harrington, Langthorne, Ngo, & Cairney, 2020; Ashton, Staresina, & Cairney, 2022; Cairney, Lindsay, Paller, & Gaskell, 2018; Durrant, Cairney, & Lewis, 2016; Gais, Lucas, & Born, 2006; Payne et al., 2012; Petzka, Zika, Staresina, & Cairney, 2023; Talamini, Nieuwenhuis, Takashima, & Jensen, 2008). Whereas the memory benefits of sleep were initially thought to be passive in nature (with sleep protecting offline memory processing from the interference arising from wakeful experience), it is now generally accepted that sleep plays an active role in maintaining memories for the long term (although see Yonelinas, Ranganath, Ekstrom, & Wiltgen, 2019). The influential *Active Systems Consolidation* framework proposes that a finely-tuned interplay between the cardinal brain rhythms of deep non-rapid eye movement sleep (i.e., slow-wave sleep; SWS) promotes the reactivation of newly formed declarative memories, and their consequent redistribution from hippocampus to neocortex for long-term storage (Born, Rasch, & Gais, 2006; Brodt, Inostroza, Niethard, & Born, 2023; Klinzing, Niethard, & Born, 2019; Rasch & Born, 2013). Supporting this view, a number of animal and human studies have shown that patterns of brain activity observed during training are replayed in SWS (Schönauer et al., 2017; Skaggs & McNaughton, 1996; Tatsuno et al., 2020; Wilson & McNaughton, 1994), and that this replay correlates with subsequent retrieval performance (Cairney, Guttesen, El Marj, & Staresina, 2018; Schreiner, Petzka, Staudigl, & Staresina, 2021; Wang et al., 2019). Moreover, neuroimaging studies in humans have demonstrated that overnight sleep reduces retrieval-related activity in the hippocampus (Takashima et al., 2009), with time spent in SWS predicting the extent of such hippocampal disengagement (Cairney, Durrant, Power, & Lewis, 2015; Takashima et al., 2006).

A potential prediction emerging from the Active Systems framework is that overnight consolidation (and thus hippocampal-to-neocortical memory transfer) should restore learning capabilities in the hippocampus (Walker, 2009). Initial support for this idea comes from studies showing that a night of sleep deprivation (relative to a normal night of sleep) impairs next-day learning (Alberca-Reina, Cantero, & Atienza, 2014; Cousins, Sasmita, & Chee, 2018; Guttesen et al., 2022; Kaida, Niki, & Born, 2015; Tempesta et al., 2016) and reduces encoding-related responses in hippocampus (Yoo, Hu, Gujar, Jolesz, & Walker, 2007). Correspondingly, more recent studies have shown that daytime naps increase hippocampal engagement (and behavioural performance) at learning, as compared to an equivalent period of wakefulness (Mander, Santhanam, Saletin, & Walker, 2011; Ong, Lau, Lee, van Rijn, & Chee, 2020). The capacity for forming new declarative memories is also linked with markers of SWS during the previous period of sleep (Antonenko, Diekelmann, Olsen, Born, & Mölle, 2013; Kaida et al., 2015; Mander et al., 2011; Ong et al., 2018; Van Der Werf et al., 2009). Taken together, this indirect evidence suggests that sleep-associated consolidation and subsequent learning may share overlapping neurocognitive mechanisms, however, no direct link between consolidation and new learning can be inferred.

In a more direct test of the hypothesis that sleep-associated memory consolidation and next-day learning are linked, Guttesen et al. (2022) examined whether the benefits of sleep for declarative memory retention were predictive of sleep-associated gains in encoding performance. Although a night of sleep (vs sleep deprivation) robustly improved memory retention and subsequent learning, there was no significant relationship between the two effects, leading to the conclusion that the capacity for encoding new declarative memories is not contingent on consolidation processes unfolding across the preceding night of sleep.

Importantly, however, Guttesen et al. (2022) used two separate tasks to quantify overnight consolidation (object locations) and next-day learning (word-image pairings), meaning the resultant declarative memories were qualitatively different from one another. Although this distinction served to mitigate any potential impact of proactive or retroactive interference between the two tasks (Brophy, Jackson, & Crowe, 2009), it might have inadvertently reduced the likelihood of detecting a relationship between consolidation and subsequent learning. More specifically, while both tasks are thought to recruit the hippocampus at encoding (Eichenbaum, 2004; Konkel & Cohen, 2009), the underlying representations might be too distinct for the consolidation of one type of memory to be linked to new learning of the other. Further work is therefore needed to fully investigate the proposed assumption of the Active Systems framework: that sleep-associated consolidation of hippocampus-dependent memories is a mechanism that supports the formation of new hippocampus-dependent memories.

In the current study, we ran two preregistered online behavioural experiments (https://osf.io/khwzm and https://osf.io/jywvz) to examine the impact of sleep on memory retention and subsequent learning, and test the hypothesis that overnight consolidation is linked to next-day learning of hippocampus-dependent memories. Note, all deviations from the preregistrations have been outlined in Table S1 and are referenced in the main text. In both experiments, participants learned a set of word pairs and were tested after a 12-h delay containing overnight sleep or daytime wakefulness (indexing memory consolidation). Participants then encoded a new set of word pairs and were tested immediately afterwards (indexing new learning). In keeping with our earlier work (Guttesen et al., 2022), we predicted that sleep would improve memory consolidation and new learning, as compared to wakefulness. Moreover, consistent with the view that sleep-associated consolidation supports next-day learning, we predicted that post-delay word pair retention would correlate positively with new word pair learning after sleep, but not after wakefulness (see preregistration deviation Table S1A).

So that we could determine whether any relationship between retention and subsequent learning is restricted to memories of the same type (i.e., word pairs) or generalises to other forms of hippocampus-dependent memory, Experiment 1 also included an index of visuospatial memory retention (which was subjected to a correlational analysis with new word pair learning). We omitted our assessment of visuospatial memory from Experiment 2 to ensure that the visuospatial task did not interfere with the overnight consolidation of our primary word pair task. Hence, across two experiments, we could conceptually replicate and extend our previous lab-based work within an online environment.

## 2 Experiment 1

### 2.1 Method

#### 2.1.1 Participants

One-hundred-and-eighty-nine participants were recruited via Prolific (https://prolific.co/) and were randomly assigned to a Sleep Group or Wake Group. As indicated by self-report, all participants were native English speakers who resided in the United Kingdom. Following standardised practices in our lab (Ashton, Harrington, Guttesen, Smith, & Cairney, 2019; Guttesen, Denis, Gaskell, & Cairney, 2024; Harrington, Ngo, & Cairney, 2021; Strachan et al., 2020), participants were instructed to abstain from alcohol and caffeine for the duration of the study, and participants in the Wake Group were instructed to refrain from napping during the retention interval. Informed consent was obtained from all participants in line with the requirements of the Research Ethics Committee of the Department of Psychology at the University of York.

Participants were required to complete two separate online sessions. As preregistered, participants were excluded from all analyses if they: failed to return for the second session (*N* = 24), did not meet the performance thresholds in our behavioural tasks (*N* = 45; see details below), napped in the Wake Group (*N* = 1) or fell outside the desired age range of 18–30 years (N = 2, see supplementary Table S2).

After exclusions, our final sample comprised 115 participants (Sleep Group *N* = 57, Wake Group *N* = 58, see Table 1 for demographic information). A power analysis based on an effect size of d = 0.57 and an alpha level of 0.05 (two-tailed) revealed that for 85 % power we would need a minimum sample of *N* = 114 (N = 57 per group). The effect size was derived from a *t*-test comparing word pair retention across a 12 h delay of either overnight sleep or daytime wakefulness, performed on data from an earlier online study (Ashton & Cairney, 2021).

**Table 1:** Participant demographics (Experiment 1).

	Sleep Group	Wake Group
N (Male/Female/Non-Binary)	57 (15/42/0)	58 (14/44/0)
Age (Years)	24.05 (± 3.32)	24.24 (± 3.40)
Sleep Duration: Typical Night (Hours)	7.52 (± 1.07)	7.63 (± 1.14)
Sleep Duration: Night Before Study (Hours)		
Pre-Delay	7.52 (± 1.05)	7.26 (± 1.11)
Post-Delay	7.25 (± 1.16)	N/A
Morning/Evening Type		
Definitely morning type (N)	3	10
Rather morning type (N)	20	13
Rather evening type (N)	22	20
Definitely evening type (N)	12	15

Age, sleep parameters, and morning/evening preference were based on self-report. Data are shown as means (± SD) unless specified otherwise. For our assessment of morning/evening preference, the distribution of responses did not differ significantly between the Sleep and Wake Groups (X^2^(3, *N* = 115) = 5.67, *p* = .13). There were significantly more participants with an overall evening preference (*N* = 69) than an overall morning preference (*N* = 46; X^2^(1, N = 115) = 4.6, *p* = .032).

#### 2.1.2 Online recruitment and randomisation

The day before the first experimental session, participants completed a screening questionnaire that included questions about demographics (age, gender, and years of education), sleep habits, and morning/evening preference. Importantly, to help control for individual differences in morning/evening preference, screening questionnaires were made available at three different times of day: 09.00 h, 15.00 h, or 21.00 h. Once these slots had been filled, we randomly assigned participants to either the Wake or Sleep Group (and to one of three word-pair counterbalancing conditions, see Materials). Participants were then sent a message informing them about the times that their sessions would begin the next day, i.e., either starting in the morning (Wake Group) or the evening (Sleep Group). Of the 449 participants who completed the screening questionnaire, 189 returned for at least the first experimental session. Although this is substantial attrition, it is important to note that the final sample comprised a reasonably even spread of participants from the three different sign-up times (09.00 h *N* = 37; 15.00 h *N* = 38; 21.00 h *N* = 40, see supplementary Table S2) with no associations between sign-up time and allocated group (X^2^ = 0.54, *p* = .764).

### 2.2 Materials

Twenty images of emotionally neutral scenes were acquired from the Nencki Affective Picture System (Marchewka, Zurawski, Jednoróg, & Grabowska, 2014) for use in the visuospatial memory task.

Ninety semantically related word pairs were acquired from the University of South Florida Word Association Norms (Nelson, McEvoy, & Schreiber, 1998) for use in the word pair task. Word pairs were divided into three lists of 30 for use in three separate recall tests. List assignment to recall test was counterbalanced across participants. The lists did not differ significantly in terms of semantic relatedness, cue length, or target length (all pairwise *p* > .05).

### 2.3 Procedure

**Fig. 1:**
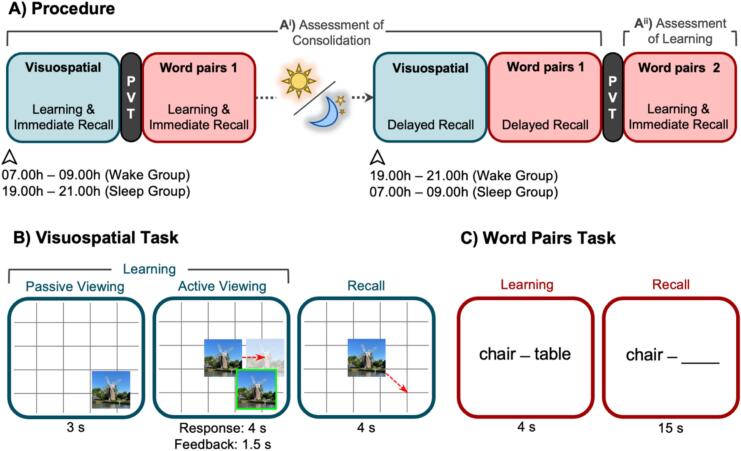
Procedures and tasks (Experiment 1). (A^i^) Procedure to assess memory consolidation. Participants encoded and were tested on the locations of 20 images. After a short Psychomotor Vigilance Task (PVT), participants encoded 60 word pairs and were tested on half of those pairs. After a 12 h delay of either overnight sleep (Sleep Group) or daytime wakefulness (Wake Group), participants were tested again on the locations of the 20 images, and were also tested on the other half of the word pairs. (A^ii^) Procedure to assess new learning. After a second PVT, participants learned 30 new word pairs and were then tested on all of these pairs. (B) Visuospatial Task. Participants completed one round of Passive Viewing, where they viewed the location of each image on a grid. Next, in the Active Viewing phase, participants moved each image to the location that they believed it had appeared during Passive Viewing and received feedback on its correct location (green frame). Active Viewing continued until participants had met the performance criterion for all images (see below) on two consecutive rounds of Active Viewing, or until they had completed 10 rounds of Active Viewing (whichever came first). The test phases followed the same procedures as one round of Active Viewing, but no feedback was provided. (C) Word Pairs Task. At encoding, participants viewed each of the word pairs. At recall, participants saw the first word of each pair (cue) and were required to type the corresponding second word (target). (For interpretation of the references to colour in this figure legend, the reader is referred to the web version of this article.)

The experimental procedure is illustrated in Fig. 1A. Participants completed two sessions (Pre-Delay and Post-Delay), which were separated by a 12 h delay containing either a night of sleep (Sleep Group) or a day of wakefulness (Wake Group). The sessions were only available to access online from 07:00–09:00 h for the morning session and 19:00–21:00 h for the evening session. The participants were required to begin the session within those two hours, after which the session links were unavailable. The study was completed using a desktop or laptop computer (smartphones and tablets were not permitted). The first session lasted ∼35 min and the second session lasted ∼20 min.

Participants completed short questionnaires to assess their chronotype, typical overnight sleep duration, and sleep duration on the night before each session. Chronotype was assessed using a single forced-choice question: *One hears about “morning” and “evening” types of people. Which one of these types do you consider yourself to be? 1) Definitely morning type; 2) Rather morning type; 3) Rather evening type; 4) Definitely evening type*.

#### 2.3.1 Assessment of consolidation

Before the sleep or wake interval (Pre-Delay session), participants completed two memory tasks: a visuospatial task (Fig. 1B) and a word pair task (Fig. 1C). Immediately after each task, participants completed a recall test that assessed their memory for the information they had just encoded. After the sleep or wake interval, participants completed additional recall tests that re-assessed their memory for the image locations and word pairs (Post-Delay session). These elements of the experiment were designed to assess memory consolidation (Figure 1A^i^). All experimental tasks were created using Pavlovia (https://pavlovia.org/).

#### 2.3.2 Visuospatial memory: Training

The visuospatial training task consisted of 20 image locations that were learned across two phases: Passive Viewing and Active Viewing. On each trial of the Passive Viewing phase, a randomly selected image was presented at a random location on a grid background for 3 s and was followed by a 0.5 s inter-stimulus interval (ISI). Participants saw all 20 images once and were instructed to memorize the image locations for a future test. On each trial of the Active Viewing phase, a randomly selected image appeared in the centre of the grid, and participants were required to move the image to the location that they believed it had appeared during the Passive Viewing phase within 4 s. The image then reappeared in a green frame at its correct location for 1.5 s (to serve as feedback, 0.5 s ISI). Active Viewing rounds continued until all 20 images had been placed within 0.42 normalized units (NU^2^) of the centre of the correct image location (referred to hereafter as the success zone) on two consecutive rounds (images for which this criterion was met were dropped from subsequent Active Viewing rounds), or until participants had completed 10 rounds of Active Viewing (whichever came first). The size of the success zone was based on Guttesen et al. (2022) laboratory study, which used a success zone of 300 × 300 pixels. We converted this value to NU^2^ to ensure compatibility across different devices (with different screen resolutions).

#### 2.3.3 Visuospatial memory: Recall

The immediate and delayed visuospatial memory tests followed the same procedures as one round of Active Viewing, with the exception that participants did not receive any feedback on the correct image locations.

#### 2.3.4 Word pair memory: Training

Participants viewed each of 60 semantically related word pairs (e.g., *chair - table*). On each trial, a randomly selected word pair was presented in the centre of the screen for 4 s, and was followed by a 1 s ISI. Participants were informed that their memory for the word pairs would be tested. To help participants remember the word pairs, they were instructed to think of a mental image or story in their mind that linked the words together.

To check whether participants were paying attention, twelve attention check trials were randomly intermixed within the word pair trials. On each attention check, a pair of randomly generated four-digit numbers (e.g., *8121–3482*) appeared in the centre of the screen and participants were instructed to press the Space Bar within 5 s. As preregistered, individuals who failed more than two attention check trials in the Pre-Delay session were not invited back for the Post-Delay session.

#### 2.3.5 Word pair memory: Test

At the immediate test, participants' memory was assessed for the first 30 word pairs that were shown in the training phase. On each trial, the first word (cue) of a randomly selected pair was presented in the centre of the screen. Participants were instructed to type the corresponding second word (target) within 15 s, and then press the “Enter” key to submit their response. As preregistered, participants who completed the word pair test but achieved <20 % correct were excluded from all analyses.

When participants were unable to recall the target word, they were instructed to type the cue word. For example, if “*chair -* ” appeared on the screen, and the participant was unable to recall the target word (i.e., *table*), they would simply type and submit the word “*chair*”. This instruction was designed to prevent participants from providing low-quality responses to complete the task more quickly (i.e., it would take as long to type the target word as it would the cue word). As preregistered, participants who submitted either a blank response or a nonsense word on >20 % of trials were not invited back for the Post-Delay session. All words that were not a perfect match with the target word were reviewed by the researchers. Nonsense words were defined as incorrect responses for which there was no clear referent in the English dictionary.

The delayed recall test was identical to the immediate recall test, except that memory was assessed for the other half of the word pairs from training (i.e., the latter half that did not feature in the immediate recall test). There was no performance criterion in the delayed test.

#### 2.3.6 Assessment of learning

After the delayed recall tests, participants learned a new set of word pairs and were immediately tested on them. This component of the experiment was designed to assess learning after the sleep/wake delay (Figure 1A^ii^). This followed identical procedures to the word pair task described above, except that it contained half as many word pairs (*N* = 30) and attention check trials (*N* = 6), and all of the word pairs were assessed in a single test phase. As before, participants were excluded if they achieved <20 % correct or submitted either a blank response or a nonsense word on >20 % of trials. As preregistered, participants were excluded if they failed >1 attention check.

#### 2.3.7 Alertness

Subjective sleepiness was assessed at the beginning of each session using the Stanford Sleepiness Scale (Hoddes, Zarcone, Smythe, Phillips, & Dement, 1973). Vigilance was assessed before each word pair task using a 3 min Psychomotor Vigilance Task (PVT; Ashton & Cairney, 2021; Ashton et al., 2022). During the PVT, participants were shown a blank grey screen. At random intervals between 2 and 10 s, a red cross appeared in the centre of the screen and participants were required to press the Space Bar as quickly as possible.

### 2.4 Data analysis

Unless stated otherwise, all data were analysed in RStudio (RStudio Team v.1.4.1717, 2021).

### 2.5 Consolidation

#### 2.5.1 Visuospatial memory

To assess the consolidation of visuospatial memories across the sleep/wake delay, we first calculated how precisely participants recalled the location of each image, separately for each recall test (i.e., immediate and delayed). Specifically, we computed an error score that reflects the distance between the correct image location (i.e., the location presented at Passive Viewing) and the location recalled by the participant at test. Next, for each image, we calculated the difference in error score between the two recall tests [immediate recall test – delayed recall test]. Finally, we calculated the average of the differences to create an overall Visuospatial Retention Index for each participant. Higher scores therefore reflect better retention (see preregistration deviation Table S1B).

#### 2.5.2 Word pair memory

We also assessed the consolidation of word pairs across the sleep/wake delay. We began by calculating the percentage of correctly recalled word pairs, separately for each recall test (i.e., immediate and delayed). Responses were considered correct if they were identical to the target word, were a plural or singular of the target word (e.g., *fungi* vs *fungus*), or were identical barring a clear and obvious spelling or typographical error (e.g., *descent* vs *desent*). Responses were considered incorrect if they were a different participle of the target word (e.g., *draw* vs *drawing*). To quantify retention across the sleep/wake delay, we calculated a Word Pair Retention Index: the difference in recall performance between the two recall tests [delayed recall test – immediate recall test]. Higher scores thus reflect better retention (see preregistration deviation Table S1C).

To investigate the effect of sleep (vs wakefulness) on consolidation, we conducted independent *t*-tests (Sleep Group vs Wake Group) on the Visuospatial Retention Index and Word Pair Retention Index and computed Cohen's d effect sizes using the pooled standard deviation estimate (R package: lsr, function: cohensD; Navarro, 2015).

### 2.6 New Learning

To assess new learning, we calculated the percentage correct in the test for the second set of word pairs (Figure 1A^ii^). This measure is referred to hereafter as the Learning Index. To investigate the effect of sleep (vs wakefulness) on learning, we conducted an independent t-test (Sleep Group vs Wake Group) on the Learning Index.

Parallel to the t-tests used to investigate consolidation and new learning, we calculated Bayes Factors (BF) to quantify evidence for the experimental and null hypotheses (R package: BayesFactor, function: ttestBF; Morey & Rouder, 2018). We used a default prior of √(2)/2 (Ly, Verhagen, & Wagenmakers, 2016) because we expected a medium effect size. Our interpretation of the BF follows the standard recommendations (Jarosz & Wiley, 2014; Jeffreys, 1961). Specifically, a BF between 1 and 3 implies anecdotal evidence, 3–10 substantial evidence, and 10–30 strong evidence.

### 2.7 Relationship between consolidation and subsequent learning

To assess the relationship between consolidation and subsequent learning, we examined the correlation between our retention indices (Visuospatial Retention Index, Word Pair Retention Index) and the Learning Index using skipped Pearson's correlations (MATLAB toolbox: Robust Correlation; Pernet, Wilcox, & Rousselet, 2013), separately for the Sleep and Wake Groups. Skipped correlations detect and ignore outliers by considering the overall structure of the data, providing accurate false positive control without loss of power. We compared the skipped correlations between groups using Zou's confidence intervals (R package: cocor; Zou, 2007). Skipped correlations were interpreted as significantly different if Zou's confidence interval did not contain zero (Zou, 2007). BF correlations were also computed (R package: BayesFactor, function: correlationBF; Morey & Rouder, 2018). We used a default prior of 1/3, as this is deemed most appropriate when addressing a novel research question (Ly et al., 2016). The outliers detected and ignored in the skipped correlations were excluded from the BF correlations.

### 2.8 Alertness

We calculated the frequency of attention lapses (trials where the participant failed to respond within 500 ms; Lim & Dinges, 2008) for each participant in each PVT task and applied these to a 2 (Group: Sleep, Wake) x 2 (Session: Pre-Delay, Post-Delay) mixed ANOVA. Because of a violation of the normality assumption, we used robust ANOVA with 20 % trimmed means (R package: walrus; Love & Mair, 2018; Mair & Wilcox, 2020).

We also assessed whether self-reported sleepiness differed between groups in either session by repeating the robust two-way mixed ANOVA with Stanford Sleepiness Scale scores as the dependent variable (these also violated the normality assumption). The ANOVA was followed up with post-hoc Bonferroni-corrected Mann-Whitney *U* tests where appropriate.

### 2.9 Results

#### 2.9.1 Baseline recall performance

There were no significant differences in performance between the Sleep and Wake Groups at the immediate recall tests (visuospatial task: t(113) = 0.81, *p* = .42, d = 0.15; word pair task: t(113) = 1.46, *p* = .15, d = 0.27; See Table 2).

**Table 2:** Memory performance (Experiment 1).

A	Visuospatial memory (error score)			
	Sleep Group		Wake Group	
	Immediate (PM)	Post-Delay (AM)	Immediate (AM)	Post-Delay (PM)
	0.38 (± 0.02)	0.49 (± 0.03)	0.35 (± 0.02)	0.55 (± 0.02)


B	Word pair memory (recall)			
	Sleep Group		Wake Group	
	Immediate (PM)	Post-Delay (AM)	Immediate (AM)	Post-Delay (PM)
	60.53 (± 2.32)	32.63 (± 2.70)	55.63 (± 2.42)	18.62 (± 2.12)

(A) Error scores in the visuospatial task (normalized units), which quantify the distance between the correct image location and the participant's recalled location. Higher scores thus reflect poorer recall accuracy. (B) The percentage of correctly recalled target words in the word pair task. Data are shown as means (± SEM).

#### 2.9.2 Sleep and memory consolidation

**Fig. 2:**
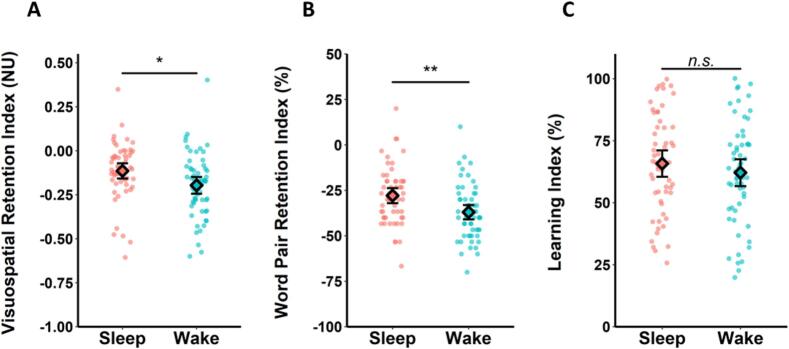
Retention and new learning (Experiment 1). Retention was better over sleep than wakefulness in the visuospatial task (A) and the word pair task (B). However, there was no significant between-group difference in subsequent word pair learning (C). Data are shown as means ± SEM. Data points represent individual participants. (**) *p* < .01; (*) *p* < .05; (n.s.) non-significant.

To test our hypothesis that overnight sleep benefits memory consolidation, we compared retention indices between the Sleep and Wake Groups. As predicted, the Visuospatial Retention Index was significantly higher in the Sleep Group than the Wake Group (t(113) = 2.54, *p* = .012, d = 0.47, BF_10_ = 3.47; Fig. 2A), as was the Word Pair Retention Index (t(113) = 3.19, *p* = .002, d = 0.60, BF_10_ = 17.29; Fig. 2B). Together, these results suggest that visuospatial and word pair memories were better retained across a night of sleep than a day of wakefulness.

#### 2.9.3 Sleep and new learning

Next, we tested our hypothesis that new learning would be better after overnight sleep than daytime wakefulness. Contrary to our prediction, the Learning Index did not differ significantly between the Sleep and Wake Groups (t(113) = 0.97, *p* = .34, d = 0.18; Fig. 2C). A complementary Bayesian analysis demonstrated substantial support for the null hypothesis (BF_01_ = 3.32). Because the memory test immediately followed learning, it is possible that participants could have retained the final few word pairs from the learning phase in their working memories (Cowan, 2010). To account for the possible contribution of working memory, the analysis was repeated after excluding the final three word pairs shown at learning. The statistical significance of the results did not change after these exclusions.

#### 2.9.4 Relationship between consolidation and new learning

**Fig. 3:**
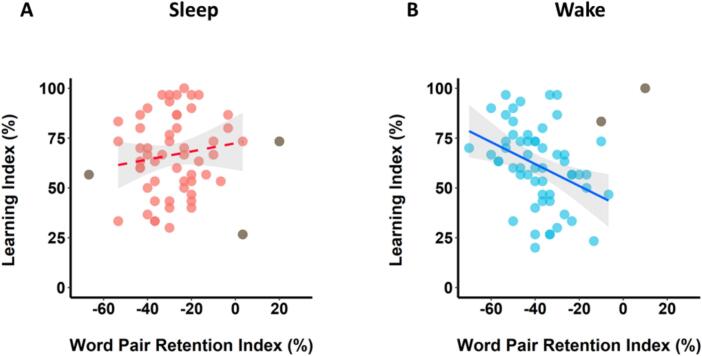
Relationship between retention and new learning (Experiment 1). (A) In the Sleep Group, there was no significant correlation between retention and subsequent learning of word pairs. (B) In the Wake Group, there was a significant negative correlation, such that greater word pair retention was associated with poorer subsequent word pair learning. Shaded areas represent 95 % confidence intervals. Individuals who were identified as outliers by the skipped correlation analysis and thus did not contribute to the relationship (see Method) are shown in grey (*N* = 3 in A, *N* = 2 in B).

To test our hypothesis that overnight consolidation benefits next-day learning, we first analysed the relationship between retention and subsequent learning of word pairs in the Sleep Group. Contrary to our prediction, there was no significant correlation between the Word Pair Retention Index and Learning Index (r-skipped = 0.13, [−0.10, 0.33] bootstrapped 95 % CI; Fig. 3A), with anecdotal support for the null hypothesis (BF_01_ = 2.14). There was also no significant correlation between the Visuospatial Retention Index and the Learning Index (r-skipped = 0.21, [−0.01, 0.41] bootstrapped 95 % CI), and the evidence for the null was anecdotal (BF_01_ = 1.13). These results suggest that overnight consolidation does not influence next-day learning, regardless of whether the to-be-learned information is qualitatively identical to or different from the consolidated information.

We also tested the relationship between retention and subsequent learning in the Wake Group. Unexpectedly, there was a significant negative correlation between the Word Pair Retention Index and the Learning Index (r-skipped = −0.37, [−0.55, −0.18] bootstrapped 95 % CI, BF_10_ = 9.81; Fig. 3B). That is, the more word pairs that participants retained across the waking delay, the poorer they were at learning new word pairs after the delay. The correlation differed significantly from the one observed in the Sleep Group (Zou's 95 % CI [0.14, 0.82]). There was no significant correlation between the Visuospatial Retention Index and the Learning Index in the Wake Group (r-skipped = 0.00, [−0.34, 0.30] bootstrapped 95 % CI; Zou's 95 % CI [−0.56, 0.16]), with substantial evidence for the null (BF_01_ = 3.36), suggesting that wakeful retention is only associated with subsequent new learning when the to-be-learned information is qualitatively identical to the retained information.

To account for the possible contribution of working memory, the analysis was repeated after excluding the final three word pairs shown at learning. These exclusions did not affect the results.

### 2.10 Alertness

For the PVT, there were more attention lapses in the Post-Delay session than the Pre-Delay session across all participants (F(1,226) = 5.09, *p* = .026; Table 3A). However, performance did not differ significantly between the Sleep and Wake Groups in either Session (main effect of Group: F(1,226) = 0.36, *p* = .55; Group x Session interaction: F(1,226) = 0.24, *p* = .63).

**Table 3:** Vigilance and sleepiness (Experiment 1).

A	Psychomotor Vigilance Task			
	Sleep Group		Wake Group	
	Pre-Delay (PM)	Post-Delay (AM)	Pre-Delay (AM)	Post-Delay (PM)
	11.20 (± 2.43) 5.12_tr_	11.49 (± 1.67) 8.69_tr_	7.37 (± 1.17) 4.98_tr_	11.41 (± 2.08) 7.28_tr_


B	Stanford Sleepiness Scale			
	Sleep Group		Wake Group	
	Pre-Delay (PM)	Post-Delay (AM)	Pre-Delay (AM)	Post-Delay (PM)
	2.97 (±0.09) 2.94_tr_	3.61 (± 0.18) 3.51_tr_	3.47 (± 0.14) 3.28_tr_	2.72 (± 0.17) 2.44_tr_

(A) The percentage of attention lapse trials in the psychomotor vigilance task. Attention lapses are trials where participants failed to respond within 500 ms. (B) Stanford Sleepiness Scale scores, which quantify subjective sleepiness. Higher scores reflect greater sleepiness. Data are shown as means (± SEM) and 20 % trimmed (_tr_) means (used by robust ANOVA).

For self-reported sleepiness (Stanford Sleepiness Scale), there was a significant Group x Session interaction (F(1,226) = 32.50, *p* = .001; Table 3B). Post-hoc tests showed that the Wake Group felt sleepier than the Sleep Group in the Pre-Delay session (W = 1198.50, *p* = .010), whereas the Sleep Group felt sleepier than the Wake Group in the Post-Delay session (W = 2341.00, *p* < .001). There was also a main effect of Group (F(1,226) = 8.90, *p* = .004; Table 3B), such that the Sleep Group felt generally sleepier than the Wake Group (despite performing better in the memory tasks). There was no main effect of Session (F(1,226) = 1.13, *p* = .29).

We conducted exploratory analyses to investigate whether individual differences in subjective sleepiness could have influenced our measures of consolidation and subsequent learning. Specifically, in both the Sleep and Wake Groups, we investigated whether the retention indices (Visuospatial Retention Index, Word Pair Retention Index) were associated with between-session changes in participant sleepiness, and whether the Learning Index was associated with sleepiness in the Post-Delay session. We used Spearman's rank correlation coefficient due to violation of the normality assumption. After Bonferroni corrections, none of the results were statistically significant (corrected *p* > .05), suggesting that changes in subjective sleepiness were unlikely to influence task performance (see Table S3). To further investigate whether within-session sleepiness contributed to memory performance, these correlations were also computed (e.g., Pre-Delay immediate memory performance and Pre-Delay sleepiness rating). None of the additional within-session correlations were statistically significant (all *p* > .218, uncorrected). We also found no significant relationships between memory performance and self-reported sleep duration during the delay (Sleep Group) or on the night before the Pre-Delay session (both groups; see supplementary Table S6).

## 3 Experiment 2

Experiment 2 was practically identical to Experiment 1, with the exception that the visuospatial memory tasks and PVTs were not included. Accordingly, there was less scope for word pair learning to be affected by interference from prior tasks, offering a potentially cleaner assessment of the relationship between overnight consolidation and next-day learning.

### 3.1 Method

#### 3.1.1 Participants

Three hundred and thirteen participants were recruited via Prolific (https://prolific.co/), completed the screening questionnaire, and were randomly assigned to a Sleep or Wake Group. Of these, 184 participants returned to complete at least the first experimental session. As preregistered, sixty-one participants were excluded from all analyses because: their demographic data was not recorded due to a technical fault (*N* = 1), they correctly recalled <20 % of the target words in the recall tests (*N* = 12), they failed to return for the Post-Delay session (*N* = 24), they failed >2 attention checks in the Pre-Delay learning task (*N* = 19), or they submitted a blank response or nonsense word on >20 % of trials in the immediate recall test (*N* = 5, not including those already excluded for failing too many attention checks). This resulted in a final sample of 123 participants (Sleep Group *N* = 58, Wake Group *N* = 65, Table 4), who comprised a relatively even spread of participants from the three different sign-up times (09.00 h *N* = 36; 15.00 h N = 36; 21.00 h *N* = 51, see supplementary Table S4) with no association between sign-up time and allocated group (X^2^ = 0.62, *p* = .732).

**Table 4:** Participant demographics (Experiment 2).

	Sleep Group	Wake Group
N (Male/Female/Non-Binary)	58 (14/44/0)	65 (17/47/1)
Age (Years)	22.69 (± 3.41)	22.86 (± 3.67)
Typical Sleep Duration (Hours)	7.52 (± 0.96)	7.31 (± 1.34)
Sleep Duration Before Study (Hours)		
Pre-Delay	7.52 (± 1.31)	6.63 (± 1.24)
Post-Delay	6.89 (± 1.17)	N/A
Morning/Evening Type		
Definitely morning type (N)	3	8
Rather morning type (N)	22	23
Rather evening type (N)	15	18
Definitely evening type (N)	18	16

Age, sleep parameters, and morning/evening preference were based on self-report. Data are shown as means (± SD) unless specified otherwise. For our assessment of morning/evening preference, the distribution of responses did not differ significantly between the Sleep and Wake Groups (X^2^(3, *N* = 123) = 2.29, *p* = .51). The number of participants with an overall evening preference (*N* = 67) did not differ significantly from the number of participants with an overall morning preference (*N* = 56; X^2^(1, N = 123) = 0.98, *p* = .32).

The inclusion criteria and participant instructions were identical to those of Experiment 1. None of the participants from Experiment 1 participated in Experiment 2, as verified by cross-referencing Prolific participant IDs. Informed consent was obtained from all participants in line with the requirements of the Research Ethics Committee of the Department of Psychology at the University of York.

### 3.2 Materials

We used the same word pairs that were used in Experiment 1. The 90 word pairs were divided into the same three lists of 30 pairs for use in three separate recall tests. The lists were counterbalanced across recall tests.

### 3.3 Procedure

We used the same procedure as Experiment 1 (Fig. 1A), with the exception that the visuospatial tasks and the PVTs were omitted. Moreover, instead of testing participants on the first/s half of the encoded word pairs in the respective immediate/delayed tests, the word pairs were randomly allocated to tests. All experimental tasks were created using Gorilla (https://gorilla.sc/). Each session lasted ∼15 min.

### 3.4 Data analysis

The statistical analysis was identical to Experiment 1. Although we did not preregister the use of Bayesian analyses or Zou's confidence intervals, these analyses were performed for consistency with Experiment 1.

### 3.5 Results

#### 3.5.1 Baseline recall performance

Performance in the immediate recall test did not differ significantly between the Sleep and Wake Groups (t(121) = 1.32, *p* = .19, d = 0.24; Table 5).

**Table 5:** Memory performance (Experiment 2).

Word Pair (Recall)			
Sleep Group		Wake Group	
Immediate (PM)	Post-Delay (AM)	Immediate (AM)	Post-Delay (PM)
53.62 (± 2.37)	31.61 (± 2.38)	58.00 (± 2.31)	25.33 (± 2.09)

The percentage of correctly recalled target words in the word pair task. Data are shown as means (± SEM).

#### 3.5.2 Sleep and memory consolidation

**Fig. 4:**
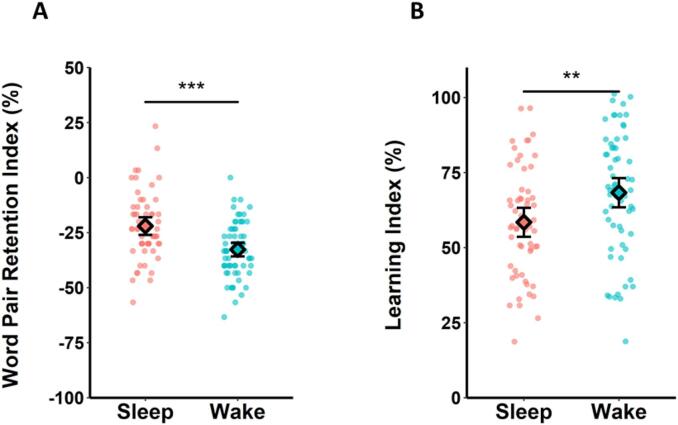
Retention and new learning (Experiment 2). (A) Word pair retention was better over sleep than wakefulness. (B) New learning (word pairs) was better after wakefulness than sleep. Data are shown as mean ± SEM. Data points represent individual participants. (***) p < .001; (**) *p* < .01.

The Word Pair Retention Index was significantly higher in the Sleep Group than the Wake Group (t(121) = 4.26, *p* < .001, d = 0.77, BF_10_ = 484.93; Fig. 4A). This result replicates the findings of Experiment 1 and suggests that sleep supported memory consolidation.

#### 3.5.3 Sleep and new learning

Contrary to expectations, the Learning Index was significantly higher in the Wake Group than the Sleep Group (t(121) = 2.87, *p* = .005, d = 0.52, BF_10_ = 7.36; Fig. 4B).

#### 3.5.4 Relationship between consolidation and new learning

**Fig. 5:**
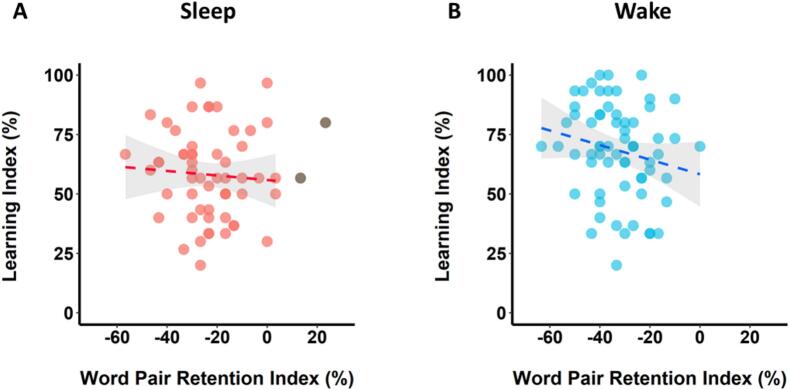
Relationship between retention and new learning (Experiment 2). There was no significant correlation between retention and new learning in the Sleep Group (A) or the Wake Group (B). Shaded areas represent 95 % confidence intervals. Individuals who were identified as outliers by the skipped correlation analysis and thus did not contribute to the relationship (see Method) are shown in grey (*N* = 2 in A, *N* = 0 in B).

Consistent with Experiment 1, there was no significant relationship between the Word Pair Retention Index and Learning Index in the Sleep Group (r-skipped = −0.07, [−0.31, 0.20] bootstrapped 95 % CI; Fig. 5A) with anecdotal evidence for the null (BF_01_ = 2.95), suggesting that overnight consolidation does not influence next-day learning.

There was also no significant relationship between the Word Pair Retention Index and Learning Index (r-skipped = −0.19, [−0.40, 0.00] bootstrapped 95 % CI; BF_10_ = 0.86; Fig. 5B) in the Wake Group, although the direction of the relationship was in keeping with Experiment 1, whereby greater retention over wakefulness was associated with poorer subsequent learning. The skipped correlations did not differ significantly between the Sleep and Wake Groups (Zou's 95 % CI [−0.23, 0.47]).

### 3.6 Alertness

Our analysis of self-reported sleepiness (Stanford Sleepiness Scale) revealed a significant Group x Session interaction (F(1,242) = 27.19, *p* = .001; Table 6). Post-hoc tests showed that the Sleep Group felt sleepier than the Wake Group in the Post-Delay session (W = 2859.50, p < .001), but there was no significant between-group difference in the Pre-Delay session (W = 1531.00, *p* = .12). There was also a main effect of Group, with the Sleep Group feeling generally sleepier than the Wake Group (F(1,242) = 11.21, *p* = .002). There was no main effect of Session (F(1,242) = 0.57, *p* = .81).

**Table 6:** Sleepiness (Experiment 2).

Stanford Sleepiness Scale			
Sleep Group		Wake Group	
Pre-Delay (PM)	Post-Delay (AM)	Pre-Delay (AM)	Post-Delay (PM)
2.69 (± 0.11) 2.67_tr_	3.67 (± 0.19) 3.56_tr_	3.20 (± 0.17) 3.00_tr_	2.35 (± 0.18) 2.03_tr_

Stanford Sleepiness Scale scores. Higher scores reflect greater sleepiness. Data are shown as means (± SEM) and 20 % trimmed (_tr_) means (used by robust ANOVA).

In exploratory analyses, we investigated the relationship between subjective sleepiness and our measures of consolidation and new learning. In the Wake Group, there was a significant correlation between the Retention Index and between-session changes in participant sleepiness (*r* = 0.37, Bonferroni corrected *p* = .008), suggesting that individuals who became sleepier across the waking delay retained more word pairs. No other significant relationships emerged (*p* > .05, see Table S5). Additionally, to investigate whether within-session sleepiness contributed to memory performance, these correlations were also computed. None of the additional within-session correlations were statistically significant (all *p* > .074, uncorrected). We also found no significant relationships between memory performance and self-reported sleep duration during the delay (Sleep Group) or on the night before the Pre-Delay session (both groups; see supplementary Table S6).

### 3.7 Napping

We did not preregister any exclusion criteria regarding unsolicited napping in Experiment 2. Accordingly, we conducted exploratory analyses to investigate whether our results were affected by participants in the Wake Group who reported that they had napped during the retention interval (*N* = 16). Excluding these participants had no impact on the overall pattern of results (see Supplementary Analyses 1 for a detailed overview of these analyses).

### 3.8 Integrated analyses

Experiments 1 and 2 were powered to detect a benefit of sleep on memory retention (Ashton & Cairney, 2021). Accordingly, we may not have had sufficient statistical power to detect an association between memory retention and new learning in the Sleep Group. To address this issue, we combined the data from both experiments and report the exploratory analyses below (Sleep Group *N* = 115, Wake Group *N* = 123).

### 3.9 Results

#### 3.9.1 Relationship between consolidation and new learning

**Fig. 6:**
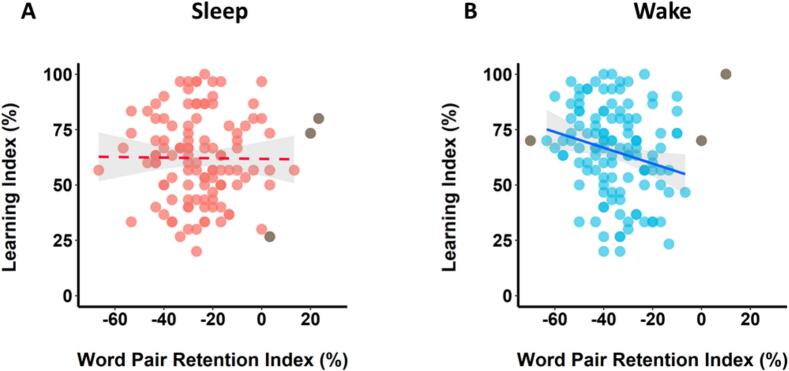
Relationship between retention and new learning (integrated analysis). (A) In the Sleep Group, there was no significant correlation between word pair retention and new learning. (B) In the Wake Group, there was a significant negative correlation, such that greater word pair retention was associated with poorer subsequent learning. Shaded areas represent 95 % confidence intervals. Individuals who were identified as outliers by the skipped correlation analysis and thus did not contribute to the relationship (see Method) are shown in grey (*N* = 3 in A, N = 3 in B).

We again observed no significant correlation between the Word Pair Retention Index and Learning Index in the Sleep Group (r-skipped = −0.01, [−0.15, 0.14] bootstrapped 95 % CI; Fig. 6A) and there was substantial evidence in favour of the null hypothesis (BF_01_ = 4.57).

There was a significant negative correlation between the Word Pair Retention Index and Learning Index in the Wake Group (r-skipped = −0.22, [−0.37, −0.07] bootstrapped 95 % CI; Fig. 6B) with substantial evidence for the alternative hypothesis (BF_10_ = 3.62), suggesting that greater retention was associated with poorer subsequent learning in these participants. The skipped correlations did not differ significantly between the Sleep and Wake Groups (Zou's 95 % CI [−0.32, 0.18]).

#### 3.9.2 Controlling for initial encoding performance

Across all participants, performance on the immediate recall test (*word pairs 1* in Fig. 1) was correlated with the Learning Index (*word pairs 2* in Fig. 1; Table 7A), demonstrating that, unsurprisingly, participants who encoded more word pairs in the Pre-Delay session also learned more word pairs in the Post-Delay session. There was also a negative correlation between immediate recall (*word pairs 1*) and the Word Pair Retention Index (for *word pairs 1*; Table 7B), suggesting that participants who learned more tended to exhibit greater rates of forgetting across the retention interval. These stable individual differences in encoding and retention could have given rise to the negative correlation between forgetting and subsequent learning observed in the Wake Group (Fig. 6B). For example, an individual scoring highly on the immediate recall test (*word pairs 1*) would have forgotten more of those word pairs after the delay (comprising a low Word Pair Retention Index), but would then also score highly on the test of new learning after the delay (*word pairs* 2, comprising a high Learning Index). These individual differences in encoding and retention might have also masked any positive effect of overnight consolidation on next-day learning in the Sleep Group.

**Table 7:** Relationship between learning and retention (integrated analysis).

	Sleep Group	Wake Group
A	r-skipped = 0.65	r-skipped = 0.65
Immediate test *(word pairs 1)* and Learning Index *(word pairs 2)*	CI = [0.55, 0.74]	CI = [0.55, 0.73]
B	r-skipped = −0.34	r-skipped = −0.59
Immediate test *(word pairs 1)* and Word Pair Retention Index *(word pairs 1)*	CI = [−0.46, −0.21]	CI = [−0.69, −0.47]
C	r-skipped = 0.62	r-skipped = 0.60
Delayed test *(word pairs 1)* and Learning Index *(word pairs 2)*	CI = [0.50, 0.73]	CI = [0.47, 0.70]

Exploratory skipped correlation (r-skipped) coefficients and confidence intervals (CI) of the relationship between performance on immediate test (*word pairs 1* in Fig. 1) and (A) Learning Index (*word pairs 2*) or (B) Word Pair Retention Index (for *word pairs 1*), and (C) delayed test (word pairs 1) and Learning Index (word pairs 2).

**Fig. 7:**
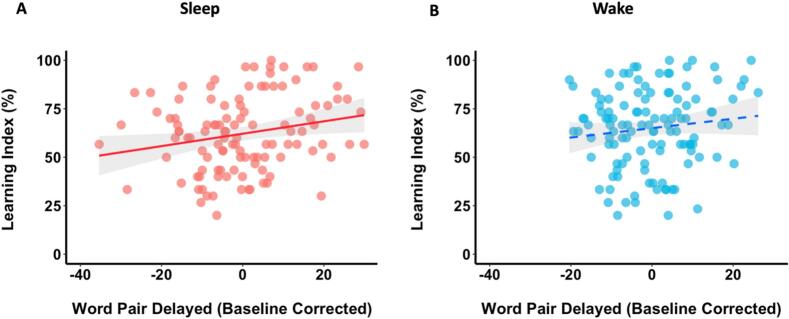
Controlling for immediate recall in the relationship between delayed recall (residuals) and new learning (integrated analysis). (A) In the Sleep Group, there was a significant correlation between delayed word pair recall and new learning after accounting for immediate word pair recall. (B) In the Wake Group, there was no significant relationship. Shaded areas represent 95 % confidence intervals.

To address these possibilities, we set out to investigate the relationship between retention and subsequent learning whilst controlling for the impact of initial learning on retention. Because our Word Pair Retention Index was calculated as the difference between performance on the immediate and delayed recall tests [delayed recall test – immediate recall test], we reasoned that it was more statistically appropriate to control for the impact of immediate recall on delayed recall (rather than on the Retention Index). Specifically, we conducted a semi-partial correlation on the relationship between performance on the delayed test (for *word pairs 1*) and the Learning Index (for *word pairs* 2) in our integrated Wake Group (excluding the same outliers as the skipped correlation) whilst controlling for the influence of immediate test performance (*word pairs 1*) on delayed test performance (*word pairs 1*, R package: ppcor, function: spcor.test; Kim, 2015). Interestingly, the negative correlation between delayed test performance and the Learning Index in the Wake Group was not significant in this exploratory analysis (*r* = 0.13, *p* = .16, Fig. 7B).

Applying the same semi-partial correlation to the integrated Sleep Group revealed a significant positive correlation between delayed test performance and subsequent learning (*r* = 0.22, *p* = .020, Fig. 7A). This suggests that overnight retention is correlated with next-day learning when pre-sleep performance is held constant. The semi-partial correlations did not differ significantly between the Sleep and Wake Groups (Zou's 95 % CI [−0.34, 0.16]).

Given the impact of immediate recall performance on memory retention, we also explored whether the learning index was different for the Sleep and Wake Groups after accounting for the immediate test. This model was significant (F(2,235) = 79.83, *p* < .001), but the Learning Index was not significantly different between the groups when controlling for immediate recall (*p* = .089).

### 3.10 Discussion

Previous work has suggested that sleep supports the consolidation and new learning of episodic declarative memories (Diekelmann & Born, 2010; Feld & Diekelmann, 2015; Walker & Stickgold, 2006). The Active Systems framework argues that episodic declarative memories are reactivated and transformed during sleep such that they become independent of hippocampus and integrated within neocortex for the long term (Born et al., 2006; Brodt et al., 2023; Klinzing et al., 2019; Rasch & Born, 2013). One potential implication of this theory is that overnight memory consolidation might support new learning in hippocampus (Walker, 2009), but empirical support for this idea is scarce. We addressed this gap in knowledge across two preregistered behavioural experiments. Overnight sleep improved memory consolidation, as compared to daytime wakefulness, but there was no benefit of sleep on new learning. In our preregistered analysis, among participants who slept there was no significant correlation between memory consolidation and subsequent learning. However, in an exploratory analysis that exploited the large N of the combined experiments and controlled for retrieval performance in the immediate pre-sleep test, a significant relationship between memory consolidation and subsequent learning did emerge in the sleep group.

Our observation that declarative memory retention was better after sleep than wakefulness is in keeping with a wide range of lab-based studies (Cairney, Guttesen, et al., 2018; Cairney, Lindsay, et al., 2018; Durrant et al., 2016; Gais et al., 2006; Guttesen et al., 2022; Payne et al., 2012; Talamini et al., 2008) and, more recently, online experiments showing that sleep supports memory consolidation (Ashton et al., 2022; Ashton & Cairney, 2021; Kroneisen & Kuepper-Tetzel, 2021; Petzka et al., 2023). The benefits of sleep for memory retention were observed for both visuospatial (Experiment 1) and word pair memories (Experiments 1 and 2), suggesting that sleep-associated consolidation influences various forms of information in the declarative domain, as observed in previous work (Schönauer, Pawlizki, Köck, & Gais, 2014). Interestingly, the effect size underlying the benefit of sleep (vs wakefulness) on word pair retention was markedly larger in Experiment 2 (d = 0.76) than in Experiment 1 (d = 0.60), potentially reflecting the absence of visuospatial training (and reduced overall memory load) in our second experiment. This is in line with a previous study finding retention benefits of sleep for lower but not higher memory loads (Feld, Weis, & Born, 2016).

It is plausible that the differences in memory retention between our sleep and wake groups could reflect passive protection from interference during overnight sleep (vs daytime wakefulness), rather than an active benefit of sleep on memory consolidation. Indeed, the Contextual Binding hypothesis argues that sleep supports memory by reducing contextual interference (Yonelinas et al., 2019). According to this view, any reactivation of memories during sleep is merely a result of residual activity from the context in which the memory was learned. If the context changes, the residual activity decreases, which contributes to forgetting. For our study, participants in the wake group went about their everyday lives during the delay between the two experimental sessions, which would likely induce contextual changes (e.g., environment, mood, etc.), whereas participants in the sleep group would have lower levels of contextual change because they slept during the delay. With this in mind, our findings may suggest that overnight sleep passively protects memory retention by reducing contextual interference.

Unexpectedly, there was no benefit of sleep for subsequent learning, with the Wake Group even outperforming the Sleep Group in Experiment 2. An exploratory analysis also showed learning did not significantly differ between the groups after accounting for baseline performance. These findings are at odds with a number of studies indicating that new learning is supported by sleep (Alberca-Reina et al., 2014; Antonenko et al., 2013; Van Der Werf et al., 2009). This discrepancy between our findings and previous work might be explained by a key difference between the experimental protocol used here and those adopted in previous studies. We used a “12–12” design, where the encoding and test sessions are separated by a 12 h interval containing overnight sleep or daytime wakefulness. Previous studies have compared learning performance after overnight sleep relative to sleep deprivation (Guttesen et al., 2022; Kaida et al., 2015; Tempesta et al., 2016; Yoo et al., 2007) or after daytime naps relative to equivalent periods of wakefulness (Mander et al., 2011; Ong et al., 2020). Thus, the benefits of sleep for new learning might not emerge when comparing performance across participants' typical sleep/wake routines.

One possibility is that time-of-day effects at encoding or retrieval counteracted the influence of intervening sleep on next-day learning. Previous work has shown that young adults tend to prefer learning in the afternoon or evening, as compared to the morning (May, Hasher, & Stoltzfus, 1993; Maylor & Badham, 2018). Likewise, participants in the current study generally considered themselves to be more “evening types” than “morning types” (see Table 1, Table 4; although note this difference was not significant in Experiment 2) and self-reported sleepiness scores indicated that participants felt more tired in the morning than the evening. Assessing the benefits of sleep for subsequent learning might be better understood using study designs that allow all sessions to take place at the same time of day (Cunningham, Stickgold, & Kensinger, 2022). It is worth noting, however, that greater subjective sleepiness was not associated with poorer task performance in either experiment. Furthermore, supplementary analyses revealed no significant differences in word-pair memory between the morning tests and evening tests (collapsed across the sleep and wake groups, Fig. S2). Altogether, these results suggest that time-of-day effects are unlikely to fully account for the null effect of sleep on new learning.

Our overarching objective was to test an indirect assumption of the Active Systems consolidation framework: that hippocampal-to-neocortical memory transfer during sleep restores hippocampal learning capabilities (Walker, 2009). Contrary to our hypothesis, our preregistered analyses showed no evidence of a relationship between overnight memory retention and next-day learning after sleep, which is in keeping with our recent lab-based findings (Guttesen et al., 2022). This result also extends our prior work by showing that the null relationship between sleep-associated consolidation and subsequent learning emerges regardless of whether the consolidated and to-be-learned materials reside in the same or different domain of declarative memory (i.e., visuospatial memories or paired associate memories).

Our preregistered analyses indicated that better memory retention across wakefulness predicted poorer subsequent learning. At first glance, this negative relationship might be driven by proactive interference: the more word pairs retained over the wake delay, the more they interfered with post-delay encoding. However, considering that this relationship did not hold up in our exploratory analyses that controlled for the impact of individual differences in learning at baseline, we think the more likely explanation is that this relationship was driven by stable individual differences in learning across the two sessions. Specifically, high performers at the immediate test (who were also better subsequent learners) were more susceptible to forgetting than low performers (who were also poorer subsequent learners) simply because they had more information to forget. Indeed, there was no significant relationship between delayed recall and new learning in the Wake Group when subsidiary analyses controlled for initial memory performance (i.e., retention prior to the waking delay).

These stable individual differences might have also prevented us from observing a positive relationship between retention and next-day learning in those participants who slept. Interestingly, a significant correlation between delayed recall and new learning did emerge in an exploratory analysis that controlled for initial memory performance at the immediate test in the integrated Sleep Group (combining Experiments 1 and 2). Thus, these findings offer some indirect support for our initial hypothesis that sleep-associated consolidation is linked to next-day learning, although it should be noted that the relationship between delayed recall and new learning did not differ significantly between the Sleep and Wake Groups. Further confirmatory studies are therefore necessary to directly assess the conditions through which overnight consolidation might influence new learning. Such work could match the level of initial encoding across individuals (e.g., training participants to a pre-determined threshold) to reduce the impact of individual differences in learning ability.

It could be argued that measuring memory retention across one night of sleep is insufficient to index behavioural changes arising from systems consolidation. Indeed, systems-level consolidation is unlikely to be completed across a matter of nights (irrespective of encoding strength) and probably takes several weeks or even months to become fully established (Dudai, 2004; Dudai, Karni, & Born, 2015). While neuroimaging studies have shown that one night of sleep is associated with major changes in the neural correlates of declarative memory retrieval (i.e., with reduced activity in hippocampus and a concomitant increase of activity in neocortex; Takashima et al., 2009), behavioural measures across one night might not be sufficient to assess emergent hippocampal-to-neocortical information transfer. Although one night of sleep might at least initiate the systems-level consolidation process, as suggested by a semi-partial correlation in our integrated data from the exploratory analysis, behavioural measures across longer delays might be better suited to detect systems-level memory consolidation.

If confirmatory studies support our exploratory finding that overnight consolidation facilitates next-day learning, it will have profound implications for our understanding of sleep's role in memory. A relationship between overnight consolidation and next-day learning is compatible with the Active Systems Consolidation framework, but it remains a relatively underdeveloped and poorly understood aspect of the theory. Strengthening the empirical basis for this link would help extend the Active Systems Consolidation model beyond its current emphasis on the strengthening of recently-acquired memories, highlighting sleep's additional role as a memory resource manager that prepares the brain for future learning. This shift in the conceptualisation of sleep-associated memory formation would treat encoding and offline consolidation as interconnected aspects of sleep's broader role in cognitive optimisation.

Such an integrated view of sleep's role in memory processing raises important mechanistic questions about the role of NREM sleep oscillations in memory resource management. If overnight memory consolidation and next-day learning are interconnected, slow oscillations—which have previously been linked to both memory consolidation (Helfrich, Mander, Jagust, Knight, & Walker, 2018; Mikutta et al., 2019) and subsequent learning (Antonenko et al., 2013; Ong et al., 2018; Van Der Werf et al., 2009)—might jointly regulate the strengthening of existing memories and the encoding of new ones. An alternative view is that overnight consolidation and next-day learning are driven by separate electrophysiological phenomena with no direct, causal link. Indeed, rodent research has shown that slow oscillations facilitate memory retention, whereas delta waves (1–4 Hz oscillations that also occur during slow-wave sleep) support forgetting (Kim, Gulati, & Ganguly, 2019). To draw causal conclusions about the role of NREM oscillations in mediating the relationship between overnight memory processing and next-day learning, future studies could boost NREM sleep oscillations using non-invasive interventions such as phase-locked auditory stimulation (Harrington & Cairney, 2021) and investigate whether the benefit of stimulation for memory consolidation is proportional to its benefit for new learning.

A recent theoretical framework has furthermore argued that memory reactivation during NREM sleep may not only promote the consolidation of certain memories, but also weaken irrelevant or unwanted memories from the same experiences (Cairney & Horner, 2024). Together with our current findings, this perspective generates further questions about how the nature of overnight memory processing (i.e., whether memories are actively strengthened or weakened during sleep) influences next-day learning. Should adaptive, sleep-associated memory consolidation go awry, learning mechanisms may likewise become impaired, giving rise to mnemonic distortions.

To conclude, we carried out two preregistered behavioural experiments to test the hypothesis that overnight consolidation paves the way for new learning of hippocampus-dependent memories. Although sleep (vs wake) improved declarative memory consolidation, there was no sleep-associated benefit on next-day learning. Our preregistered analyses revealed no correlation between overnight retention and subsequent learning, although a significant relationship did emerge in exploratory analyses that controlled for performance in the pre-sleep test. These data suggest that a night of sleep contributes to how well we remember newly formed memories, but not necessarily how well we form new memories the next day, when compared to a day awake.

## CRediT authorship contribution statement

**Anna á V. Guttesen:** Writing – original draft, Visualization, Software, Methodology, Investigation, Funding acquisition, Formal analysis, Data curation, Conceptualization. **Marcus O. Harrington:** Writing – original draft, Visualization, Validation, Software, Methodology, Investigation, Formal analysis, Conceptualization. **M. Gareth Gaskell:** Writing – review & editing, Supervision, Conceptualization. **Scott A. Cairney:** Writing – review & editing, Supervision, Project administration, Methodology, Funding acquisition, Conceptualization.

## Declaration of competing interest

The authors have no conflicts of interest to declare.

## Data Availability

Study data and analysis scripts can be retrieved here: https://osf.io/qbeh3/, pre-registrations: https://osf.io/khwzm & https://osf.io/jywvz.

## Appendix A Supplementary data

Supplementary material Image 1

## References

[bb0005] Alberca-Reina E., Cantero J.L., Atienza M. (2014). Semantic congruence reverses effects of sleep restriction on associative encoding. Neurobiology of Learning and Memory.

[bb0010] Antonenko D., Diekelmann S., Olsen C., Born J., Mölle M. (2013). Napping to renew learning capacity: Enhanced encoding after stimulation of sleep slow oscillations. The European Journal of Neuroscience.

[bb0015] Ashton J.E., Cairney S.A. (2021). Future-relevant memories are not selectively strengthened during sleep. PLoS One.

[bb0020] Ashton J.E., Harrington M.O., Guttesen A.Á.V., Smith A.K., Cairney S.A. (2019). Sleep preserves physiological arousal in emotional memory. Scientific Reports.

[bb0025] Ashton J.E., Harrington M.O., Langthorne D., Ngo H.-V.V., Cairney S.A. (2020). Sleep deprivation induces fragmented memory loss. Learning & Memory.

[bb0030] Ashton J.E., Staresina B.P., Cairney S.A. (2022). Sleep bolsters schematically incongruent memories. PLoS One.

[bb0035] Born J., Rasch B., Gais S. (2006). Sleep to remember. Neuroscientist.

[bb0040] Brodt S., Inostroza M., Niethard N., Born J. (2023). Sleep—A brain-state serving systems memory consolidation. Neuron.

[bib391] Brophy L.M., Jackson M., Crowe S.F. (2009). Interference effects on commonly used memory tasks. Archives of clinical neuropsychology.

[bb0045] Cairney S.A., Durrant S.J., Power R., Lewis P.A. (2015). Complementary roles of slow-wave sleep and rapid eye movement sleep in emotional memory consolidation. Cerebral Cortex.

[bb0050] Cairney S.A., Guttesen A.Á.V., El Marj N., Staresina B.P. (2018). Memory consolidation is linked to spindle-mediated information processing during sleep. Current Biology.

[bb0055] Cairney S.A., Horner A.J. (2024). Forgetting unwanted memories in sleep. Trends in Cognitive Sciences.

[bb0060] Cairney S.A., Lindsay S., Paller K.A., Gaskell M.G. (2018). Sleep preserves original and distorted memory traces. Cortex.

[bb0065] Cousins J.N., Sasmita K., Chee M.W.L. (2018). Memory encoding is impaired after multiple nights of partial sleep restriction. Journal of Sleep Research.

[bb0070] Cowan N. (2010). The magical mystery four: How is working memory capacity limited, and why?. Current Directions in Psychological Science.

[bb0075] Cunningham T.J., Stickgold R., Kensinger E.A. (2022). Investigating the effects of sleep and sleep loss on the different stages of episodic emotional memory: A narrative review and guide to the future. Frontiers in Behavioral Neuroscience.

[bb0080] Diekelmann S., Born J. (2010). The memory function of sleep. Nature Reviews. Neuroscience.

[bb0085] Dudai Y. (2004). The neurobiology of consolidations, or, how stable is the engram?. Annual Review of Psychology.

[bb0090] Dudai Y., Karni A., Born J. (2015). The consolidation and transformation of memory. Neuron.

[bb0095] Durrant S.J., Cairney S.A., Lewis P.A. (2016). Cross-modal transfer of statistical information benefits from sleep. Cortex.

[bb0100] Eichenbaum H. (2004). Hippocampus: Cognitive processes and neural representations that underlie declarative memory. Neuron.

[bb0105] Feld G.B., Diekelmann S. (2015). Sleep smart—Optimizing sleep for declarative learning and memory. Frontiers in Psychology.

[bb0110] Feld G.B., Weis P.P., Born J. (2016). The limited capacity of sleep-dependent memory consolidation. Frontiers in Psychology.

[bb0115] Frankland P.W., Bontempi B. (2005). The organization of recent and remote memories. Nature Reviews. Neuroscience.

[bb0120] Gais S., Lucas B., Born J. (2006). Sleep after learning aids memory recall. Learning & Memory.

[bb0125] Guttesen A.á.V., Denis D., Gaskell M.G., Cairney S.A. (2024). Delineating memory reactivation in sleep with verbal and non-verbal retrieval cues. Cerebral Cortex.

[bb0130] Guttesen A.á.V., Gareth Gaskell M., Madden E.V., Appleby G., Cross Z.R., Cairney S.A. (2022). Sleep loss disrupts the neural signature of successful learning. Cerebral Cortex.

[bb0135] Harrington M.O., Cairney S.A. (2021). Sounding it out: Auditory stimulation and overnight memory processing. Current Sleep Medicine Reports.

[bb0140] Harrington M.O., Ngo H.-V.V., Cairney S.A. (2021). No benefit of auditory closed-loop stimulation on memory for semantically-incongruent associations. Neurobiology of Learning and Memory.

[bb0145] Helfrich R.F., Mander B.A., Jagust W.J., Knight R.T., Walker M.P. (2018). Old brains come uncoupled in sleep: Slow wave-spindle synchrony, brain atrophy, and forgetting. Neuron.

[bb0150] Hoddes E., Zarcone V., Smythe H., Phillips R., Dement W.C. (1973). Quantification of sleepiness: A new approach. Psychophysiology.

[bb0155] Jarosz A.F., Wiley J. (2014). What are the odds? A practical guide to computing and reporting Bayes factors. The Journal of Problem Solving.

[bb0160] Jeffreys H. (1961).

[bb0165] Kaida K., Niki K., Born J. (2015). Role of sleep for encoding of emotional memory. Neurobiology of Learning and Memory.

[bb0170] Kim J., Gulati T., Ganguly K. (2019). Competing roles of slow oscillations and Delta waves in memory consolidation versus forgetting. Cell.

[bb0175] Kim S. (2015). Ppcor: An R package for a fast calculation to semi-partial correlation coefficients. Communications for Statistical Applications and Methods.

[bb0180] Klinzing J.G., Niethard N., Born J. (2019). Mechanisms of systems memory consolidation during sleep. Nature Neuroscience.

[bb0185] Konkel A., Cohen N.J. (2009). Relational memory and the hippocampus: Representations and methods. Frontiers in Neuroscience.

[bb0190] Kroneisen M., Kuepper-Tetzel C.E. (2021). Using day and night–scheduling retrieval practice and sleep. Psychology Learning and Teaching.

[bb0195] Lim J., Dinges D.F. (2008). Sleep deprivation and vigilant attention. Annals of the New York Academy of Sciences.

[bb0200] Love J., Mair P. (2018).

[bb0205] Ly A., Verhagen J., Wagenmakers E.-J. (2016). Harold Jeffreys’s default Bayes factor hypothesis tests: Explanation, extension, and application in psychology. Journal of Mathematical Psychology.

[bb0210] Mair P., Wilcox R. (2020). Robust statistical methods in R using the WRS2 package. Behavior Research Methods.

[bb0215] Mander B.A., Santhanam S., Saletin J.M., Walker M.P. (2011). Wake deterioration and sleep restoration of human learning. Current Biology.

[bb0220] Marchewka A., Zurawski Ł., Jednoróg K., Grabowska A. (2014). The Nencki affective picture system (NAPS): Introduction to a novel, standardized, wide-range, high-quality, realistic picture database. Behavior Research Methods.

[bb0225] Marr D., Willshaw D., McNaughton B., Vaina L. (1971). From the retina to the neocortex: Selected papers of David Marr (pp. 59–128). Birkhäuser Boston.

[bb0230] May C.P., Hasher L., Stoltzfus E.R. (1993). Optimal time of day and the magnitude of age differences in memory. Psychological Science.

[bb0235] Maylor E.A., Badham S.P. (2018). Effects of time of day on age-related associative deficits. Psychology and Aging.

[bb0240] McClelland J.L., McNaughton B.L., O’Reilly R.C. (1995). Why there are complementary learning systems in the hippocampus and neocortex: Insights from the successes and failures of connectionist models of learning and memory. Psychological Review.

[bb0245] Mikutta C., Feige B., Maier J.G., Hertenstein E., Holz J., Riemann D., Nissen C. (2019). Phase-amplitude coupling of sleep slow oscillatory and spindle activity correlates with overnight memory consolidation. Journal of Sleep Research.

[bb0250] Morey R.D., Rouder J.N. (2018). BayesFactor: Computation of Bayes factors for common designs. R package version 0.9.12-4.2. https://CRAN.R-project.org/package=BayesFactor.

[bb0255] Navarro D. (2015). https://learningstatisticswithr.com.

[bb0260] Nelson D.L., McEvoy C.L., Schreiber T.A. (1998). The University of South Florida word association, rhyme, and word fragment norms. http://www.usf.edu/FreeAssociation/.

[bb0265] Ong J.L., Lau T.Y., Lee X.K., van Rijn E., Chee M.W.L. (2020). A daytime nap restores hippocampal function and improves declarative learning. Sleep.

[bb0270] Ong J.L., Patanaik A., Chee N.I.Y.N., Lee X.K., Poh J.-H., Chee M.W.L. (2018). Auditory stimulation of sleep slow oscillations modulates subsequent memory encoding through altered hippocampal function. Sleep.

[bb0275] Payne J.D., Tucker M.A., Ellenbogen J.M., Wamsley E.J., Walker M.P., Schacter D.L., Stickgold R. (2012). Memory for semantically related and unrelated declarative information: The benefit of sleep, the cost of wake. PLoS One.

[bb0280] Pernet C.R., Wilcox R.R., Rousselet G.A. (2013). Robust correlation analyses: False positive and power validation using a new open source matlab toolbox. Frontiers in Psychology.

[bb0285] Petzka M., Zika O., Staresina B.P., Cairney S.A. (2023). Better late than never: Sleep still supports memory consolidation after prolonged periods of wakefulness. Learning & Memory.

[bb0290] Rasch B., Born J. (2013). About sleep’s role in memory. Physiological Reviews.

[bb0295] RStudio Team v.1.4.1717 (2021). http://www.rstudio.com.

[bb0300] Schönauer M., Alizadeh S., Jamalabadi H., Abraham A., Pawlizki A., Gais S. (2017). Decoding material-specific memory reprocessing during sleep in humans. Nature Communications.

[bb0305] Schönauer M., Pawlizki A., Köck C., Gais S. (2014). Exploring the effect of sleep and reduced interference on different forms of declarative memory. Sleep.

[bb0310] Schreiner T., Petzka M., Staudigl T., Staresina B.P. (2021). Endogenous memory reactivation during sleep in humans is clocked by slow oscillation-spindle complexes. Nature Communications.

[bb0315] Skaggs W.E., McNaughton B.L. (1996). Replay of neuronal firing sequences in rat hippocampus during sleep following spatial experience. Science.

[bb0320] Squire L.R., Cohen N.J., Nadel L. (1984). The medial temporal region and memory consolidation: A new hypothesis. Memory Consolidation: Psychobiology of Cognition.

[bb0325] Strachan J.W.A., Guttesen A.Á.V., Smith A.K., Gaskell M.G., Tipper S.P., Cairney S.A. (2020). Investigating the formation and consolidation of incidentally learned trust. Journal of Experimental Psychology. Learning, Memory, and Cognition.

[bb0330] Takashima A., Nieuwenhuis I.L.C., Jensen O., Talamini L.M., Rijpkema M., Fernández G. (2009). Shift from hippocampal to neocortical centered retrieval network with consolidation. The Journal of Neuroscience.

[bb0335] Takashima A., Petersson K.M., Rutters F., Tendolkar I., Jensen O., Zwarts M.J., Fernández G. (2006). Declarative memory consolidation in humans: A prospective functional magnetic resonance imaging study. Proceedings of the National Academy of Sciences of the United States of America.

[bb0340] Talamini L.M., Nieuwenhuis I.L.C., Takashima A., Jensen O. (2008). Sleep directly following learning benefits consolidation of spatial associative memory. Learning & Memory.

[bb0345] Tatsuno M., Malek S., Kalvi L., Ponce-Alvarez A., Ali K., Euston D.R., McNaughton B.L. (2020). Memory reactivation in rat medial prefrontal cortex occurs in a subtype of cortical UP state during slow-wave sleep. Philosophical Transactions of the Royal Society B.

[bb0350] Tempesta D., Socci V., Coppo M., Dello Ioio G., Nepa V., De Gennaro L., Ferrara M. (2016). The effect of sleep deprivation on the encoding of contextual and non-contextual aspects of emotional memory. Neurobiology of Learning and Memory.

[bb0355] Van Der Werf Y.D., Altena E., Schoonheim M.M., Sanz-Arigita E.J., Vis J.C., De Rijke W., Van Someren E.J.W. (2009). Sleep benefits subsequent hippocampal functioning. Nature Neuroscience.

[bb0360] Walker M.P. (2009). The role of sleep in cognition and emotion. Annals of the New York Academy of Sciences.

[bb0365] Walker M.P., Stickgold R. (2006). Sleep, memory, and plasticity. Annual Review of Psychology.

[bb0370] Wang B., Antony J.W., Lurie S., Brooks P.P., Paller K.A., Norman K.A. (2019). Targeted memory reactivation during sleep elicits neural signals related to learning content. The Journal of Neuroscience.

[bb0375] Wilson M.A., McNaughton B.L. (1994). Reactivation of hippocampal ensemble memories during sleep. Science.

[bb0380] Yonelinas A.P., Ranganath C., Ekstrom A.D., Wiltgen B.J. (2019). A contextual binding theory of episodic memory: Systems consolidation reconsidered. Nature Reviews. Neuroscience.

[bb0385] Yoo S.-S., Hu P.T., Gujar N., Jolesz F.A., Walker M.P. (2007). A deficit in the ability to form new human memories without sleep. Nature Neuroscience.

[bb0390] Zou G.Y. (2007). Toward using confidence intervals to compare correlations. Psychological Methods.

