# Supplementary material for: Does overnight memory consolidation support next-day learning?
Source: Cognition. 2025 Nov;264:106241. doi: 10.1016/j.cognition.2025.106241 (PMC12332499; doi:10.1016/j.cognition.2025.106241)
Supplement: Supplementary file 1 — Supplementary material [file mmc1.docx]

**Does overnight memory consolidation support next-day learning?**

Supplementary Materials

**Table S1. Preregistrations deviations and justifications (Experiments 1 & 2)**

|  | **Hypotheses** | | |
| --- | --- | --- | --- |
|  | **Preregistration** | **Manuscript** | **Justification** |
| **A** | “The word-pair decay index will be negatively correlated with the word-pair learning index” | “…post-delay word pair **retention** would correlate **positively** with new word pair learning after sleep…” | By replacing decay with retention, we could predict a positive correlation which we thought was more theoretically intuitive. |
|  | **Methods** | | |
|  | **Preregistration** | **Manuscript** | **Justification** |
| **B** | Visuospatial decay index = [Error score at test 2 – Error score at test 1] | “…the difference in error score between the two recall tests **[immediate recall test – delayed recall test]** …Visuospatial **Retention** Index for each participant.” | To ease understanding (i.e., higher scores = better retention), we swapped the order of Retention Index subtraction. This change yields statistically identical results aside from the sign change. |
| **C** | “Word-pair decay index, calculated as: [Percentage of correctly recalled A-Bimm pairs ─ Percentage of correctly recalled A-Bdel pairs]” | “…Word Pair **Retention** Index: the difference in recall performance between the two recall tests **[delayed recall test – immediate recall test]**.” | This was the same justification as above. |

**Table S2. Randomisation and exclusion numbers (Experiment 1)**

|  | **Number of participants per initial recruitment time (final sample only)** | | | | | | | | |  |
| --- | --- | --- | --- | --- | --- | --- | --- | --- | --- | --- |
|  | | | 09.00h | | 15.00h | | 21.00h | | | |
| Sleep Group | | | 19 | | 17 | | 21 | | | |
| Wake Group | | | 18 | | 21 | | 19 | | | |
|  | | **Number of participants excluded per exclusion criterion** | | | | | |  |  |  |
|  | | | Did not return for Session 2 | Failed attention criteria/blank responses | | Memory performance  < 20 % | | | Other | |
| Sleep Group | | | 13 | 16 | | 6 | | | 1 | |
| Wake Group | | | 11 | 18 | | 5 | | | 2 | |

Final sample refers to participants included in the final analysis. The “Other” category refers to participants (N=2) where their age was outside the required range or they reported napping (Wake Group, N=1).

**Table S3. Relationship between subjective sleepiness and memory (Experiment 1)**

| **A** | **Visuospatial Retention Index and Stanford Sleepiness Scale score change** | | |
| --- | --- | --- | --- |
|  |  | Spearman’s rho | p-value (Bonferroni corrected) |
|  | Sleep Group | .08 | .547 |
|  | Wake Group | .34 | .009 (.054) |
| **B** | **Word Pair Retention Index and Stanford Sleepiness Scale score change** | | |
|  |  | Spearman’s rho | p-value |
|  | Sleep Group | -.03 | .800 |
|  | Wake Group | .11 | .403 |
| **C** | **Word Pair Learning Index and Stanford Sleepiness Scale score at Post-Delay** | | |
|  |  | Spearman’s rho | p-value (Bonferroni corrected) |
|  | Sleep Group | -.12 | .390 |
|  | Wake Group | -.31 | .018 (.108) |

Spearman’s correlation results between Stanford Sleepiness Scale score change [Post-Delay – Pre-Delay] and (A) Visuospatial Retention Index and (B) Word Pair Retention Index. A higher Stanford Sleepiness Scale score change means that participants reported feeling sleepier in the Post-Delay session than Pre-Delay session, with a positive relationship suggesting higher memory retention with increasing reported sleepiness. (C) Spearman’s correlation between Stanford Sleepiness Scale score in the Post-Delay session and the Word Pair Learning Index. A negative relationship suggests poorer learning with more reported sleepiness. P-values are reported uncorrected with Bonferroni corrected values reported in brackets, if relevant.

**Table S4. Randomisation and exclusion numbers (Experiment 2)**

|  | **Number of participants per initial recruitment time (final sample only)** | | | | | | | | |  |
| --- | --- | --- | --- | --- | --- | --- | --- | --- | --- | --- |
|  | | | 09.00h | | 15.00h | | 21.00h | | | |
| Sleep Group | | | 15 | | 18 | | 25 | | | |
| Wake Group | | | 21 | | 18 | | 26 | | | |
|  | | **Number of participants excluded per exclusion criterion** | | | | | |  |  |  |
|  | | | Did not return for Session 2 | Failed attention criteria/blank responses | | Memory performance  < 20 % | | | Other | |
| Sleep Group | | | 16 | 18 | | 4 | | | 1 | |
| Wake Group | | | 8 | 6 | | 8 | | | 0 | |

Final sample refers to participants included in the final analysis. The “Other” category refers to a participant where the demographic data were not recorded.

**Table S5. Relationship between subjective sleepiness and memory (Experiment 2)**

| **A** | **Word Pair Retention Index and Stanford Sleepiness Scale score change** | | |
| --- | --- | --- | --- |
|  |  | Spearman’s rho | p-value (Bonferroni corrected) |
|  | Sleep Group | -.13 | .315 |
|  | Wake Group | .37 | .002 (.008) |
| **B** | **Word Pair Learning Index and Stanford Sleepiness Scale score at Post-Delay** | | |
|  |  | Spearman’s rho | p-value |
|  | Sleep Group | -.00 | .973 |
|  | Wake Group | -.04 | .744 |

(A) Spearman’s correlation results between Stanford Sleepiness Scale score change [Post-Delay – Pre-Delay] and Word Pair Retention Index. A higher Stanford Sleepiness Scale score change means that participants reported feeling sleepier in the Post-Delay session than Pre-Delay session, with a positive relationship suggesting higher memory retention with increasing reported sleepiness. (B) Spearman’s correlation between Stanford Sleepiness Scale score in the Post-Delay session and the Word Pair Learning Index. A negative relationship suggests poorer learning with more reported sleepiness. P-values are reported uncorrected with Bonferroni corrected values reported in brackets, if relevant.

**Supplementary analyses 1. Excluding participants who reported napping (Experiment 2)**

Within the 16 participants from the Wake Group who reported napping during the delay between the two experimental sessions, the average reported time spent napping was 131.25 (± 143.42) min (mean ± SD). This value was inflated by three individuals who reported napping for ≥ 300 min. Without these participants, the average time spent napping was 69.23 min (± 45.68 SD).

To investigate whether our results were affected by participants in the Wake Group who napped during the delay, we repeated the main analyses without including these participants. Firstly, the Retention Index was still significantly greater in the Sleep Group as compared to the Wake Group (t(105) = 3.79, p < .001, d = 0.74; Figure S1A). Secondly, the Learning Index was still significantly greater in the Wake Group relative to the Sleep Group (t(105) = 2.43, p = .017, d = 0.47; Figure S1B). Thirdly, there was still no significant correlation between the Retention Index and Learning Index in the Wake Group (r-skipped = -0.17, [-0.43, 0.11] bootstrapped 95% CI; Figure S1C). Finally, the skipped correlations still did not differ significantly between groups (Zou’s 95% CI [-0.60, 0.15]). In sum, these results clearly demonstrate that non-compliance with our request to refrain from napping by 16 participants in the Wake Group did not influence our key findings.


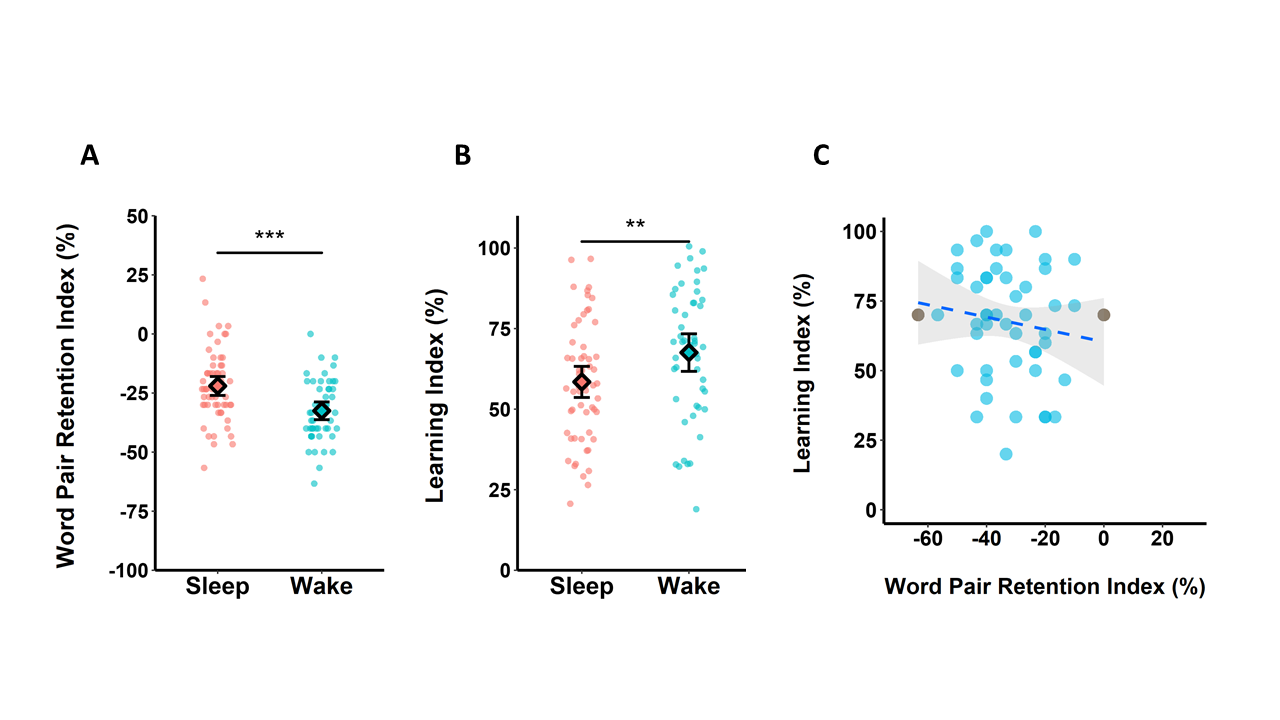


**Figure S1. Experiment 2 results after excluding participants who reported napping.** (A) Word pair retention was better over sleep than wakefulness. (B) New learning (word pairs) was better after wakefulness than sleep. (C) In the Wake Group, there was no significant correlation between retention and new learning. In A, B, and C, data points represent individual participants. In A and B, data are shown as mean ± SEM. (***) p < .001; (**) p < .01. In C, shaded areas represent 95% confidence intervals. Individuals who were identified as outliers by the skipped correlation analysis and thus did not contribute to the relationship (see Method) are shown in grey (N = 2).

**Supplementary analyses 2. Sleep duration and memory performance (Experiments 1 & 2)**

We explored whether self-reported sleep duration was linked to memory performance but found no significant relationships (Table S6).

**Table S6. Relationship between sleep duration and memory performance**

| **A** | **Sleep duration and Immediate Word Pair performance (Experiment 1)** | | |
| --- | --- | --- | --- |
|  |  | Spearman’s rho | p-value |
|  | Sleep Group | -.14 | .295 |
|  | Wake Group | -.07 | .606 |
| **B** | **Sleep duration and Immediate Word Pair performance (Experiment 2)** | | |
|  |  | Spearman’s rho | p-value |
|  | Sleep Group | -.16 | .227 |
|  | Wake Group | -.09 | .463 |
| **C** | **Sleep duration and Learning Index (Sleep Groups only)** | | |
|  |  | Spearman’s rho | p-value |
|  | Experiment 1 | .12 | .377 |
|  | Experiment 2 | -.15 | .276 |

Spearman’s correlation results between memory performance and self-reported sleep duration A & B) before the first session and C) between the sessions for the Sleep Groups (uncorrected).

**Supplementary analyses 3. Time-of-day differences in memory performance (Experiments 1 & 2)**


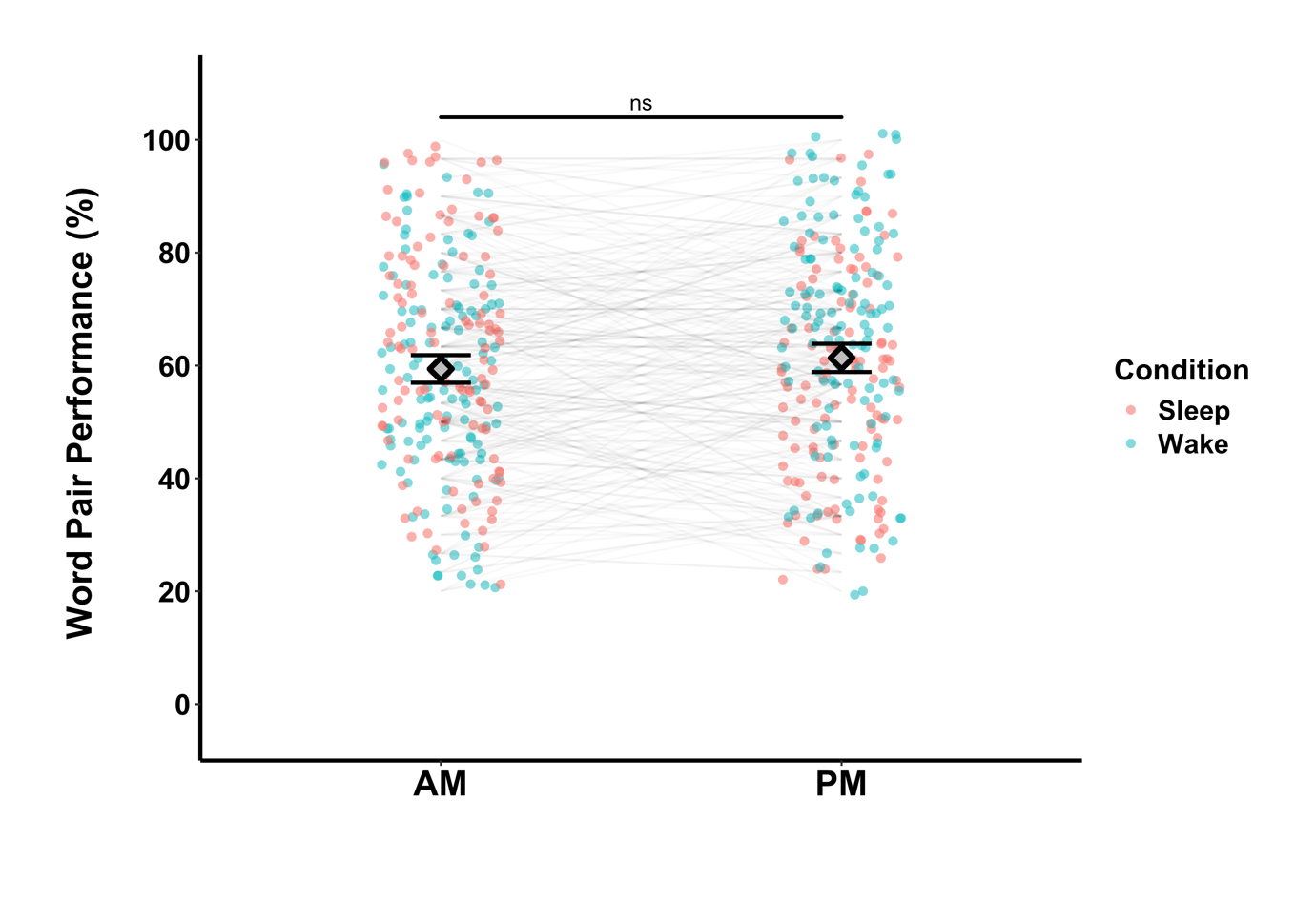


**Figure S2. Word Pair Performance in the morning and evening.** A paired-samples t-test, grouping pre-delay immediate performance in the wake condition with post-delay immediate performance (i.e., learning index) in the sleep condition (AM group) and comparing it to the PM group (i.e., post-delay immediate performance in wake condition and pre-delay immediate performance in the sleep condition) revealed no significant difference in memory performance between the time-of-day groupings (t(237)=1.71, p = .090, d=.10).
